# Era of Generalist Conversational Artificial Intelligence to Support Public Health Communications

**DOI:** 10.2196/69007

**Published:** 2025-01-20

**Authors:** Emre Sezgin, Ahmet Baki Kocaballi

**Affiliations:** 1 The Abigail Wexner Research Institute at Nationwide Children’s Hospital Columbus, OH United States; 2 College of Medicine The Ohio State University Columbus, OH United States; 3 School of Computer Science University of Technology Sydney Sydney Australia

**Keywords:** messaging apps, public health communication, language models, artificial intelligence, AI, generative AI, conversational AI

## Abstract

The integration of artificial intelligence (AI) into health communication systems has introduced a transformative approach to public health management, particularly during public health emergencies, capable of reaching billions through familiar digital channels. This paper explores the utility and implications of generalist conversational artificial intelligence (CAI) advanced AI systems trained on extensive datasets to handle a wide range of conversational tasks across various domains with human-like responsiveness. The specific focus is on the application of generalist CAI within messaging services, emphasizing its potential to enhance public health communication. We highlight the evolution and current applications of AI-driven messaging services, including their ability to provide personalized, scalable, and accessible health interventions. Specifically, we discuss the integration of large language models and generative AI in mainstream messaging platforms, which potentially outperform traditional information retrieval systems in public health contexts. We report a critical examination of the advantages of generalist CAI in delivering health information, with a case of its operationalization during the COVID-19 pandemic and propose the strategic deployment of these technologies in collaboration with public health agencies. In addition, we address significant challenges and ethical considerations, such as AI biases, misinformation, privacy concerns, and the required regulatory oversight. We envision a future with leverages generalist CAI in messaging apps, proposing a multiagent approach to enhance the reliability and specificity of health communications. We hope this commentary initiates the necessary conversations and research toward building evaluation approaches, adaptive strategies, and robust legal and technical frameworks to fully realize the benefits of AI-enhanced communications in public health, aiming to ensure equitable and effective health outcomes across diverse populations.

## Introduction

Health communication and information dissemination are essential for global risk mitigation during public health emergencies. The pandemic highlighted the necessity for effective worldwide communication networks in support of the public health agencies (Center for World Health Organization [WHO] and Global Public Health Intelligence Network), and furthermore, the need for more integrated and global systems for timely warnings and responses to health crises [1]. For this purpose, messaging services have been vital tools for disseminating information, monitoring disease spread, and promoting informed health decisions [2]. Historically, SMS- and app-based text messaging platforms have long been recognized for their potential to deliver scalable health interventions for diverse populations, exemplified by RCTs on substance abuse intervention and promoting the COVID-19 pandemic vaccination [3,4]. These examples illustrate the established value of messaging services in engaging populations and promoting health behaviors.

The emergence of artificial intelligence (AI) has brought innovative and personalized methods in health communications, with chatbots and AI-driven messaging services presenting personalized, timely, and internet-based health interventions [5]. More specifically, conversational artificial Intelligence (CAI) interventions have exhibited significant positive effects on behavior change, such as smoking cessation, healthy eating, sleep quality, and physical activity [6,7]. CAI models have emerged to complete tasks through natural language using rule-based, hybrid, or unsupervised learning models [8,9]. However, as CAI has been widely used for personalized health intervention, a public health perspective has yet to be studied.

During the pandemic, the emerging value of CAI has been observed as being a scalable, easy-to-use, and accessible dissemination tool [10-12]. In addition, the long-term value of CAI through chatbot tools and voice assistants in public health management has been emphasized [13,14]. A widely observed implementation was a WHO-deployed WhatsApp (Meta) chatbot, as a preventative mechanism, that disseminated critical health information to millions globally, providing multilingual support and real-time updates to address misinformation [15]. However, none of the intelligent systems covered earlier has envisioned the impact of generative AI at scale where it can lead to highly accessible, decentralized, and scalable implementations. Recent applications of widely adapted generative AI models, large language models (LLMs), presented evidence of the effectiveness of generated responses to answer public health questions [16,17], contributing to the trend of using AI to augment the impact of public health interventions. Envisioning the future, its impact could exponentially grow by wide-scale adoption and implementation across the population by its integration into our daily communication tools (ie, messaging apps). We hypothesize that such LLM-based CAI services (namely, “Generalist CAI” from hereon) through messaging apps, the most commonly used tools on a daily basis, may overtake other means of internet-based information seeking and public information dissemination and sharing mechanisms, toward improving public health communications.

Unlike previous implementations of CAI before the wide adoption of LLMs, Generalist CAI models rely on generative AI (ie, LLMs) or foundation models that are trained on extensive and diverse datasets to perform a myriad of natural language tasks with human-like interaction and a broad understanding across various domains, including health care. These models transcend the limitations of their predecessors, which were constrained to specific tasks, by exhibiting proficiency in varied domains, from casual dialogue to expert-level engagements. These models’ broad applicability is underpinned by their capacity to comprehend and respond in a human-like manner across different conversational contexts. As such, generalist CAI models are not only poised to enhance user interactions but are also likely to supplant multiple task-specific agents (multiagents), heralding a shift toward more unified and contextually aware conversational AI systems in diverse applications. Table 1 outlines the key features of generalist AI.

**Table 1:** Key features and descriptions of generalist conversational artificial intelligence (CAI) applications.

Key features	Descriptions
Natural language understanding and generation	They can understand a broad range of user prompts that can be expressed in various different ways, allowing a higher level of flexibility and ease-of-use.
Generalist	They can converse about a large range of topics and transition between them smoothly.
Dialog management	They can excel in multiturn conversations more effectively than their rule-based alternatives with the ability to respond to follow-up questions.
Emotional intelligence	They can detect subtle cues in language that indicate a user’s emotional state, allowing the chatbot to provide empathetic responses.
Personalization	Generalist CAI can extract user profile information and use it to generate tailored responses effectively.
Multilingual support	They can support multiple languages, making them accessible to a wider audience.
Scalability	They can handle an unlimited number of queries simultaneously, providing instant information on a wide range of public health issues.

## The Advantage of Generalist CAI in Messaging Services

AI has already transformed the ways in which individuals seek health information. Today, we observe that generalist CAI or LLM applications (eg, chatGPT) simplify access to health advice [18], by breaking down barriers associated with traditional web searches.

The next step appears to be messaging tools with generalist CAI assistance, like WhatsApp, Signal (Signal Technology Foundation), iMessage (Apple Inc.), and Telegram (Telegram FZ-LLC Telegram Messenger Inc), which would offer a “familiar” platform to billions of people, with the potential to improve access to health information and services. Messaging apps’ simplicity in terms of user interface and the use of natural language inherently reduce digital literacy and accessibility barriers. Therefore, messaging services with generalist CAI can surpass previous implementations of task-specific CAI services regarding capabilities, user access and adoption, lower barriers to use and provide personalization through self-learning and conversation adaptation (Table 1). For example, instead of just providing preprogrammed answers, a generalist CAI can engage in a dynamic dialogue to understand a user’s specific concerns, clarify ambiguities, and provide tailored advice based on the conversation’s context. Furthermore, mobile device-based dialogue services (eg, automatic speech recognition and text-to-speech) further improve the usability of the service through natural language conversations. This means, in a public health emergency, the widespread use of apps like iMessage and WhatsApp (billions of active users daily) can facilitate immediate access to personalized health advice, circumventing barriers to accessing urgent public health information. The integration with generalist CAI, as seen in Meta AI’s LLM-powered generalist CAI or personal assistant, positions these services in a favorable spot as a convenient tool for information seeking and health communication, potentially offering equitable access to personalized health information across diverse populations. To further extend these opportunities, small language models (eg, Phi-3) [19] can be leveraged to build capable generalist CAI for individuals in remote areas or those with limited access to internet bandwidth, who could still benefit from text-based interactions with the CAI.

## Envisioning Generalist CAI Use in Public Health Emergencies

**Figure 1:**
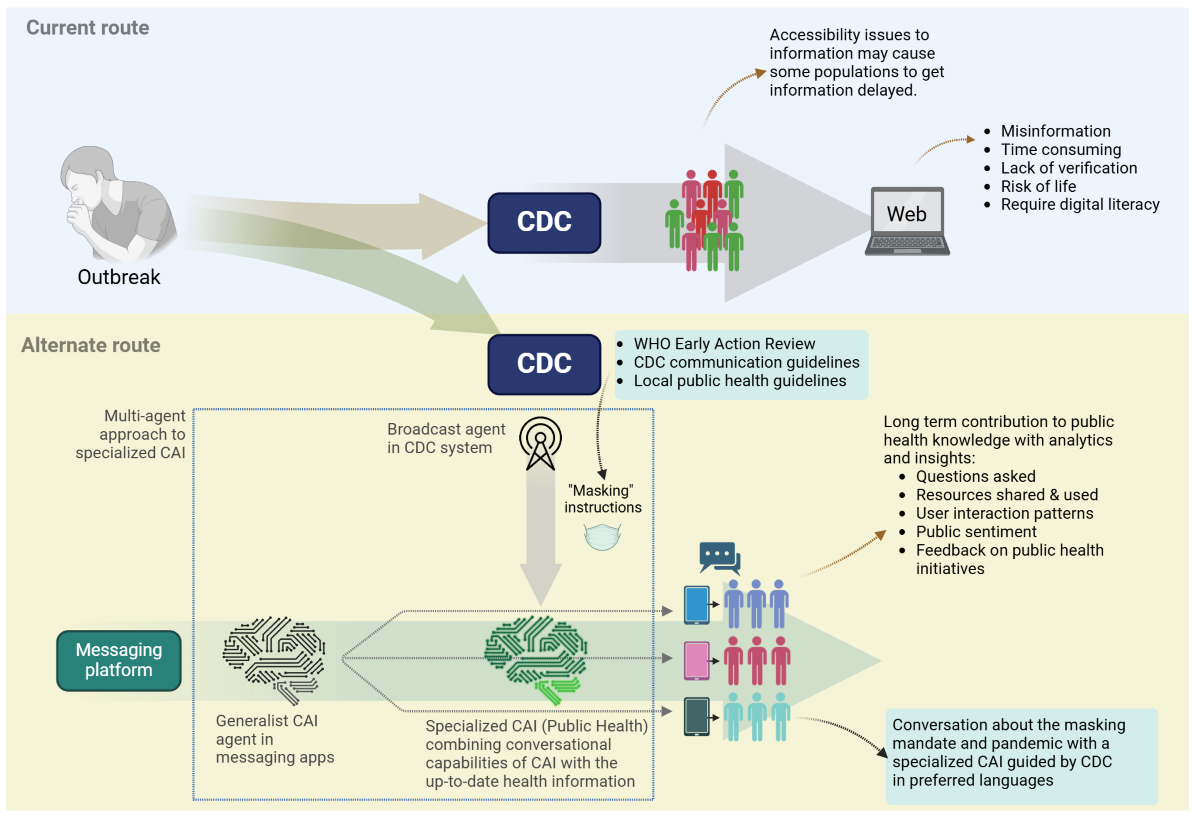
Current practice and the proposed alternative routes for public health communications during an outbreak CAI: conversational artificial intelligence; CDC: Centers for Disease Control and Prevention; WHO: World Health Organization.

As the AI tools and applications are rapidly growing and immersing in our daily lives, it is necessary to plan and strategize for the use of generalist CAI services in public health emergencies. Given the fact that the current LLMs and CAI are trained on publicly available data on a massive scale with less attention to information quality or specific domains like health care, we need to approach cautiously in promoting safe and reliable information pipelines over these messaging platforms for public health information sharing, communication, and dissemination. To improve CAI accuracy and reliability in public health communications, a multiagent approach could be used [20,21]. This includes specialized AI agents collaborating, some provide general health information, while others ensure compliance with health guidelines. We envision the integration of generalist CAI assistants into public health messaging services through such a multiagent approach to streamline future interventions. Figure 1 illustrates a 2-agent arrangement where a CAI agent in a messaging app uses the public health information provided by the Centers for Disease Control and Prevention (CDC) to compose its response.

## Public Health Emergency Case: the COVID-19 Pandemic

During the COVID-19 pandemic, we observed that the dissemination of comprehensive and accurate information is crucial for educating the public and combating the pandemic [12]. This period highlighted the potential of basic chatbot applications in enhancing public health communications, especially in diverse and low-resource settings. As aforementioned, WHO released a chatbot over WhatsApp providing up-to-date COVID-19 information in multiple local languages extending across several low- and middle-income countries [15]. In Nigeria, an SMS-based chatbot, offered by the Nigeria Centre for Disease Control, with support from UNICEF (United Nations International Children’s Emergency Fund), provided localized guidance and timely information about pandemic [22]. Similarly, India released an app with chatbot features to engage rural and urban Indian residents for the COVID-19 pandemic safety practices and checking symptoms, updates, and access helplines [23]. These implementations demonstrate the scalability and potential of digital conversational intervention in public health emergencies. With generalist CAI, personalized public health intervention can be achieved, bridging communication gaps and supporting public health agencies during emergencies.

A strategic plan for effective communication during a health crisis is required, leveraging a multiagent approach. In a partnership between the public health agencies (eg, WHO and CDC) and technology providers, we may create an information dissemination pipeline during a public health crisis (Figure 1). For instance, by adhering to CDC’s guidelines for communication and dissemination and using only vetted information sources [24], generalist CAI agents can be designed to deliver messages accurately, empathetic, and action oriented. CDC-defined key elements for developing outbreak-related messages include expressing empathy, outlining clear actions, delineating what is known and unknown, explaining public health actions and their rationale, committing to ongoing communication, and guiding the public on where to find reliable information [24]. For example, regarding the need to “outline clear actions,” a specialized agent could be programmed to ensure that every informational response includes actionable steps the user can take. This framework could be used to prompt the agents and ensure that the generated messages are fine-tuned to deliver intended messages toward not only informing and educating but also to build trust and encourage compliance with health advisories. Furthermore, aligning with WHO’s early action review guidelines that are designed to optimize early detection of public health emergencies, such agents could be deployed to intervene timely and tailored to the target audience in compliance with the local or national guidelines across the globe [25]. Ideally, a trained generalist CAI acting as a public health support agent can be simultaneously activated across the populations, including low-resource countries, rural and urban areas, served in any preferred language through text or speech. In the long term, such agents can contribute to the knowledge of public health agencies and the data they provide can help learn more about the effectiveness of communications with real-time feedback from the public. Future research should focus on developing frameworks that facilitate the integration of multiagent CAI systems into existing public health infrastructures. Some of the viable approaches could be through interoperability with electronic health records, collaboration with public health databases and registries, and using standardized communication protocols like Fast Health care Interoperability Resources [26], embedding CAI agents into communication platforms preferred by health professionals, and establishing governance mechanisms to ensure ethical and efficient operation [27,28].

Multimedia Appendix 1 for our brief experiments and observations toward the opportunities and limitations with current generalist CAI applications in response to some of the emerging public health problems including pandemic response, unmet social needs, mental health support, and vaccinations.

## Risks and Ethical Considerations

The deployment of generalist CAI assistants in messaging apps presents multifaceted challenges, including risks of bias, misinformation, hallucinations, and ethical pitfalls, which have been observed earlier with Snapchat’s personal assistant [29]. The models behind these CAI assistants pose a critical challenge due to inherent biases arising from skewed or incomplete training data, flawed algorithmic design, or the reinforcement of societal prejudices [30]. These biases risk distorting health communications and exacerbating health disparities [31,32]. For example, an earlier study [33] revealed that a widely used health care algorithm exhibited significant racial bias by predicting costs instead of health needs, leading to unequal care allocation for Black patients. Such bias could pose a risk for generalist CAI providing less comprehensive (or even inaccurate) information to individuals from underrepresented racial or ethnic backgrounds. In addition, a culturally sensitive and inclusive design is also important to mitigate the risk of inherent biases that may exist in the training data of generalist CAI. These issues underscore the importance of accountability, fairness, equity, and regulatory oversight [34]. Moreover, ethical concerns extend beyond privacy to include user autonomy and the transparent use of data, necessitating clear guidelines and user consent. To support user autonomy, implementing verification mechanisms and ensuring the source data originating from authoritative health organizations can help maintain the trustworthiness of the AI-generated information. One step further, a multiagent approach can help control the CAI behavior regarding user location and profile, ensuring that advice and data handling procedures comply with the local rules and regulations. Even though messaging services are one of the most accessible and used communication technologies, the FCC Affordable Connectivity Program or similar global programs can be used to address the digital divide and affordability issues [35]. While some messaging apps offer end-to-end encryption, overall, the lack of governance or medico-legal compliance (eg, HIPAA [Health Insurance Portability and Accountability Act], HITECH [Health Information Technology for Economic and Clinical Health] or GDPR [General data protection regulation] rule on processing sensitive data requirements for public health information), unless integrated with telehealth services, raises significant privacy concerns, similar to those of standard text messaging services. Technical standards and ethical frameworks should be developed to ensure AI systems are transparent and fair [36]. Further considerations are outlined in Table 2.

**Table 2:** Technical challenges and potential solutions in deploying generalist conversational artificial intelligence (CAI).

Challenge	Description	Potential solutions
Language diversity	Developing CAI systems that can understand and generate responses in multiple languages and dialects is complex. Language nuances, slang, and regional expressions can hinder accurate communication [37].	Multilingual training: Incorporate extensive multilingual datasets during training. Local expertise collaboration: Partner with local linguists and community experts. Adaptive learning: Implement adaptive learning algorithms based on user interactions.
Technological infrastructure	Variability in technological infrastructure, such as outdated devices or limited computational resources, can impede effective CAI deployment [38].	Lightweight models: Develop optimized, lightweight, and specialized AI models. Edge computing: use edge computing to perform data processing closer to the user. Offline capabilities: Incorporate offline functionalities where possible.
Internet connectivity	In many low-resource settings, unreliable or limited internet access can restrict the usability and accessibility of CAI-powered messaging services [39].	Data-efficient communication: Design CAI systems requiring minimal data usage. SMS or voice integration: Integrate CAI functionalities with SMS-based platforms or text-to-voice calls. Caching and preloading: Implement caching strategies to store essential information locally [40].
Integration with existing systems	Integrating CAI with existing public health information systems and workflows can be technically demanding [41].	API^a^ development: Develop robust APIs for smooth integration. Modular architecture: Adopt a modular system architecture. Standardization: Promote standardized protocols and data formats for interoperability.
Data privacy and security	Ensuring the privacy and security of user data is paramount, especially when dealing with sensitive health information [42].	Encryption and access controls: Establish strict access controls, authentication mechanisms, and end-to-end encryption. Compliance frameworks: Adhere to international and local data protection regulations (eg, GDPR^b^, HIPAA^c^). Small local models: Support the development of smaller models that can be run locally (eg, Mistral 7B and Microsoft Phi-3) [43].
Bias and cultural sensitivity	AI algorithms can inadvertently conserve social prejudices and cultural insensitivities present in training data, leading to biased or inappropriate responses [44].	Diverse training data: Curate datasets that represent diverse populations and cultural contexts [45]. Bias audits: Regularly conduct bias assessments and audits of CAI models [46]. Inclusive design practices: Incorporate principles of inclusive design to ensure cultural relevance and sensitivity [47].
Stakeholder engagement	Engaging community stakeholders is essential to ensure that CAI systems meet the needs of diverse populations and promote equitable outcomes [48].	Participatory co-design: Involve community members in the design and development process. Feedback mechanisms: Establish channels for continuous feedback from users. Collaborative partnerships: Partner with local organizations and leaders to guide CAI development and deployment.

^a^API: application programming interface.

^b^GDPR: General data protection regulation.

^c^HIPAA: Health Insurance Portability and Accountability Act.

The contentious nature of AI governance and accreditation of service providers for multiagent AI services might require a legal infrastructure as much as a technical one, to reduce perceived legal risks and liabilities with government agencies accrediting private sector tools. However, the recent initiative by the Biden Administration to form task forces aimed at shaping policies for AI in health care signals a promising direction for overcoming these hurdles in the United States [49], suggesting that improvements in the medicolegal landscape could pave the way for safer AI implementations in public health communication [50]. Internationally, regulatory bodies are also beginning to take similar steps, aiming for a cohesive global response to AI challenges [51]. It is crucial to address how these technologies might disproportionately affect marginalized groups, ensuring inclusive and equitable AI development. The long-term societal impacts, such as the erosion of public trust through AI missteps, must also be considered in developing sustainable AI strategies. Engaging a broad spectrum of stakeholders in AI discussions can enhance the legitimacy and effectiveness of governance structures. This evolving scenario highlights the critical need for a balanced approach to harnessing AI’s potential while addressing its ethical, legal, and social challenges.

## Conclusions and Future Directions

AI-enhanced messaging apps hold significant promise for advancing public health by improving access to health information, supporting health behavior change, and addressing diverse community needs. Their scalability and adaptability have already demonstrated impact during public health crises, such as the COVID-19 pandemic, where CAI systems facilitated rapid and multilingual information dissemination. Generalist CAI, with its ability to handle diverse conversational tasks and adapt to user needs with its human-like interaction capability, represents a transformative opportunity to create equitable and accessible public health communication tools. However, realizing their full potential requires addressing critical challenges, including biases in training data, risks of misinformation, ethical concerns around equity and transparency, and the need for robust legal and technical frameworks. To unlock these opportunities, AI messaging services must be developed and continually updated to address evolving requirements on fairness, accountability, and culturally sensitive design, ensuring they uphold the principles of reliability, equity, and justice in public health.

Key research priorities should include evaluating the efficacy of multiagent systems, understanding user interaction and trust dynamics, addressing biases, assessing impacts on health equity, and exploring innovative applications beyond basic information delivery. Observational studies can help identify how individuals engage with CAI systems and guide improvements in public health applications. To ensure equitable access, public health agencies should advocate for culturally sensitive and linguistically diverse CAI systems. Collaborations with local organizations and the establishment of global repositories of vetted health information are critical to minimizing misinformation and aligning CAI outputs with public health guidelines. Eventually, public health agencies and researchers can create a roadmap for leveraging CAI effectively and equitably, paving the way for a transformative approach to public health communication. Continued research on user engagement and the optimization of AI models for public health is essential to fully leverage the capabilities of these ubiquitous tools.

## Appendix

Multimedia Appendix 1 Examples of generalist CAI (conversational artificial intelligence) responses to public health issues.

## Abbreviations

AI: artificial intelligence
CAI: conversational artificial intelligence
CDC: Centers for Disease Control and Prevention
GDPR: General data protection regulation.
HIPAA: Health Insurance Portability and Accountability Act
HITECH: Health Information Technology for Economic and Clinical Health
LLM: large language model
UNICEF: United Nations International Children’s Emergency Fund
WHO: World Health Organization
