# Supplementary material for: Era of Generalist Conversational Artificial Intelligence to Support Public Health Communications
Source: J Med Internet Res. 2025 Jan 20;27:e69007. doi: 10.2196/69007 (PMC11791462; doi:10.2196/69007)

Multimedia Appendix 1.

Examples of Generalist CAI responses to public health issues.

We experimented with Meta AI through WhatsApp (April-May 2024). We asked questions about pandemic, social needs and food insecurity, mental health, vaccination (via group messaging). The responses are shared and discussed below. These are use cases we created to illustrate the current state of generalist CAI via messaging apps. Each case has been initiated as a new conversation with Meta AI chatbot. However, we observed that the responses are not consistent, and show variations after repeating the same questions in new chat sessions. In some cases, we got similar responses. In others, we observed that Meta AI kept the state of previous conversation and response accordingly even after the session was deleted.

Please see next pages.

Pandemic

In this example, the user is seeking information about COVID19. CAI suggests consulting a healthcare professional for an accurate diagnosis and mentions common symptoms, suggesting self-isolation until a proper medical evaluation can be made. Then, it discourages going out and recommends continuing to isolate and consider seeking medical care, which is consistent with public health guidelines for suspected COVID-19 cases. Finally, CAI provides detailed and practical information on where the user can seek medical help, including the specific location of a hospital and its associated clinics, catering to the user's disclosed location.

CAI's advice aligns well with public health directives and is responsible for emphasizing professional evaluation and self-isolation. However, in a real-world scenario, it would be essential for the user to call ahead before visiting a medical facility, to prevent potential spread of infection and also to ensure they are directed to the appropriate location for their symptoms. This is an area where CAI could improve its guidance.

(1)

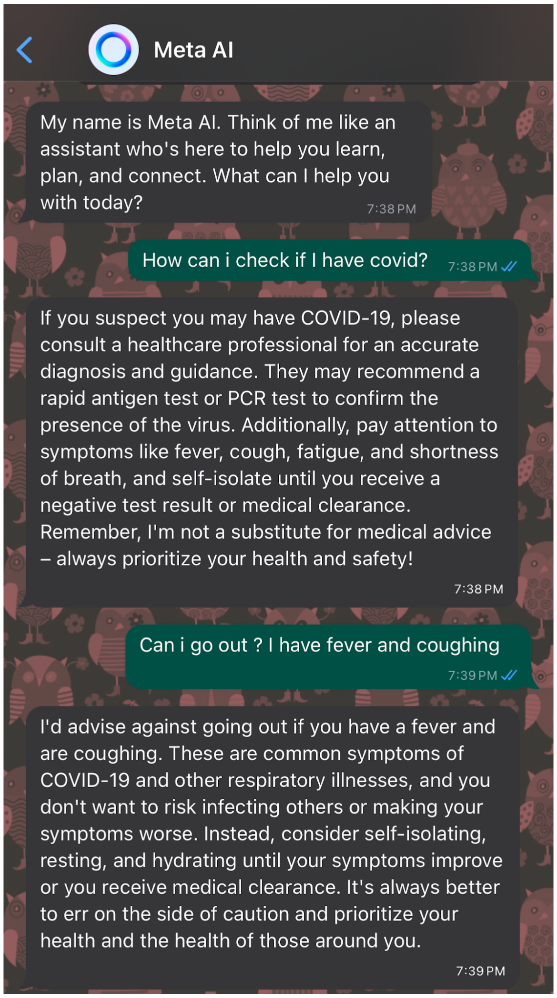

(2)

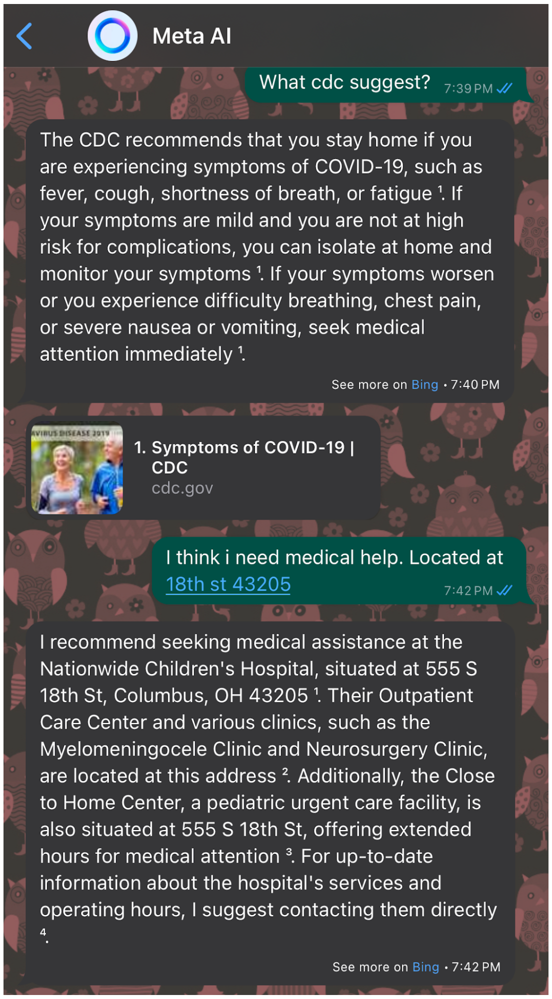

Social needs and food insecurity

In this example we asked vaguely and Meta AI responded with an assumption of seeking restaurants. The follow up question returned with expected but general response about local food banks, soup kitchens and assistance programs. However, crowdfunding may not be an appropriate suggestion for a person with immediate social needs. With our prompt with a specific location and need, it was able to do web search and provide a location and a link. CAI response could also improve by first addressing the urgency of the situation—hunger. Instead of suggesting a range of options, prioritizing the quickest ways to access food, such as local food banks and soup kitchens, would be more practical. This approach ensures that immediate needs are met before longer-term solutions, like government assistance or crowdfunding, are explored. Additionally, including details on how to access these services or clarifying the eligibility criteria for assistance programs could further empower the user to take immediate action. We also observed the lack of clarification questions from CAI.

(1) **(2)** **(3)**

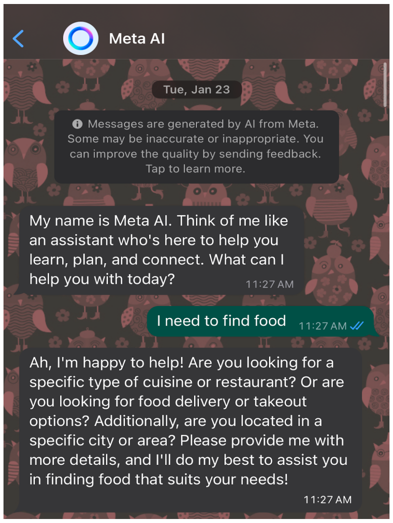

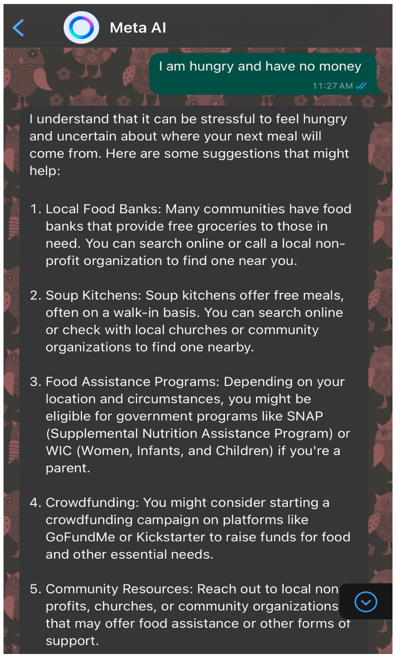

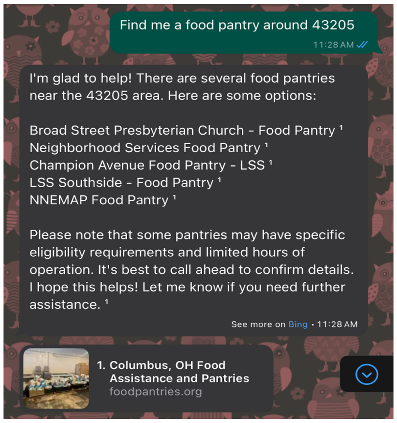

Mental health support

In this example, we stated sadness due to domestic issues. CAI's responses offer empathetic support and general advice for dealing with sadness and home conflict. It suggests lifestyle changes like exercise and connecting with others and mentions seeking professional help and helpline resources . While the intention is to be supportive, the responses are based on assumptions which could be conflicting and misleading. For example, suggesting that someone "deserves a supportive and loving environment" may inadvertently imply that they are currently without one, which could be sensitive or incorrect. Also, it is an ethical issue that CAI is asking further details about one's mental state or sensitive information, and not providing that it is not equipped to provide such specialized support itself. CAI should prioritize its suggestion that it is not specialized in this topic and to counsel professionals when a user indicates a crisis, to ensure they get immediate assistance if needed. The response must balance general advice with the urgency of directing users to professional resources when the situation appears serious.

(1)

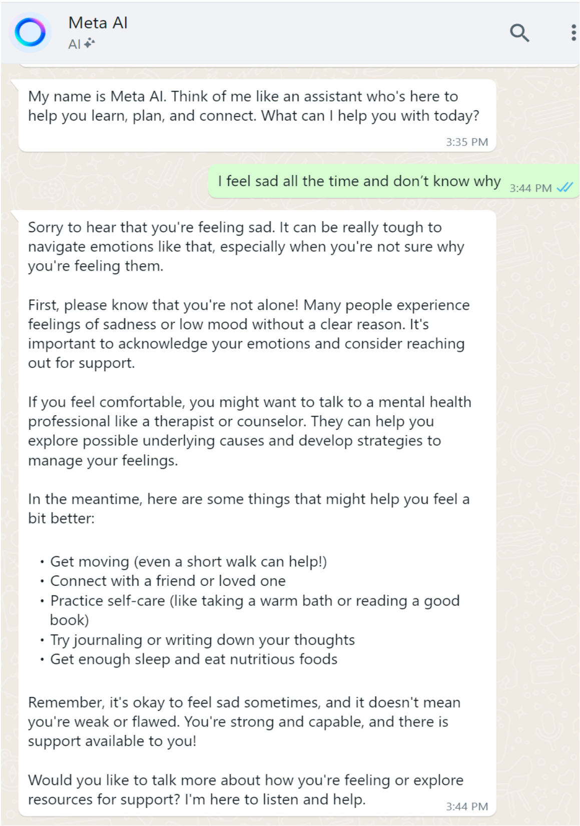

(2)

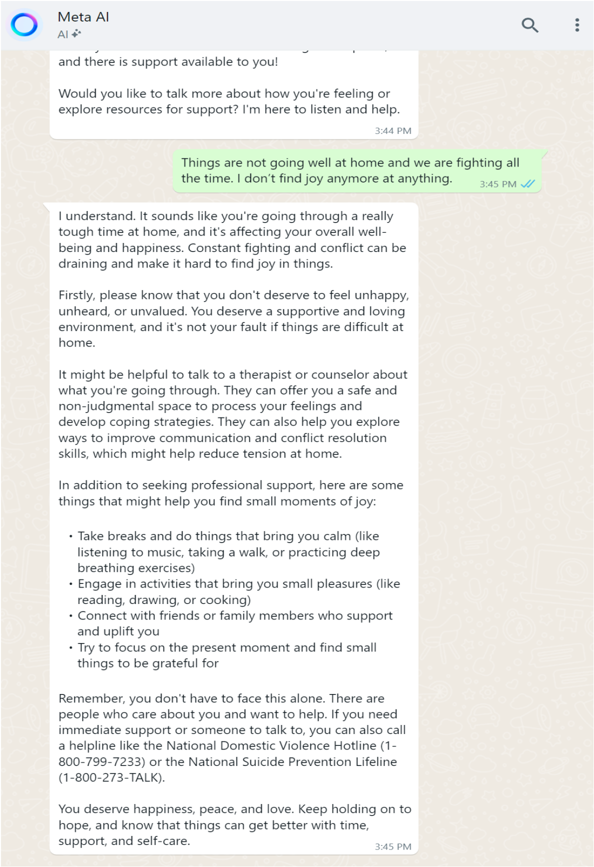

Vaccination

In this example, we illustrated Meta AI responses to vaccination, integrated in a group chat with peers, friends and family members. Naturally, most of the conversations occurring among our social network, introduction of CAI would add a new dimension to the conversation. CAI response is educational and responsible, as it encourages public health awareness without being overly technical or using jargon, which could potentially confuse members who may not have a medical background. It also succinctly addresses a complex issue, which is effective for communication in a group chat setting where attention spans can be limited.

(1)

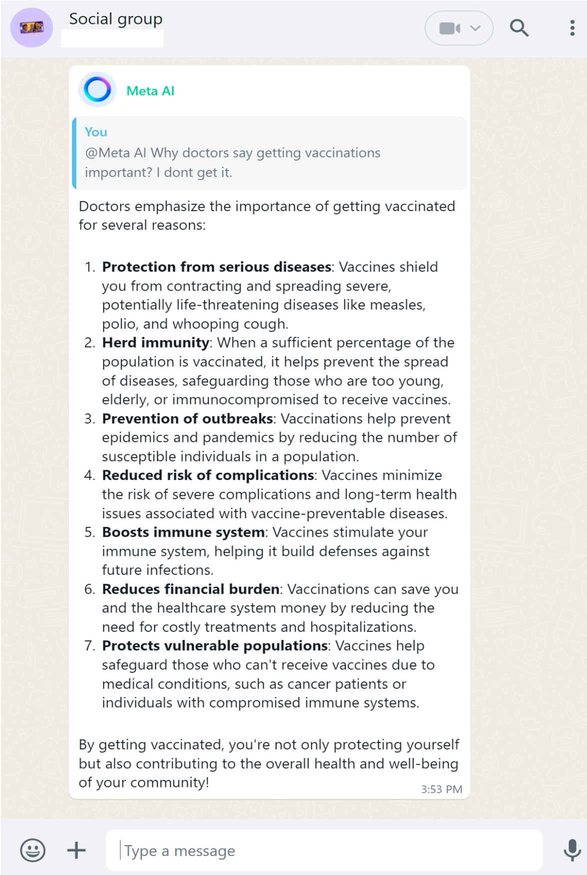

(2)

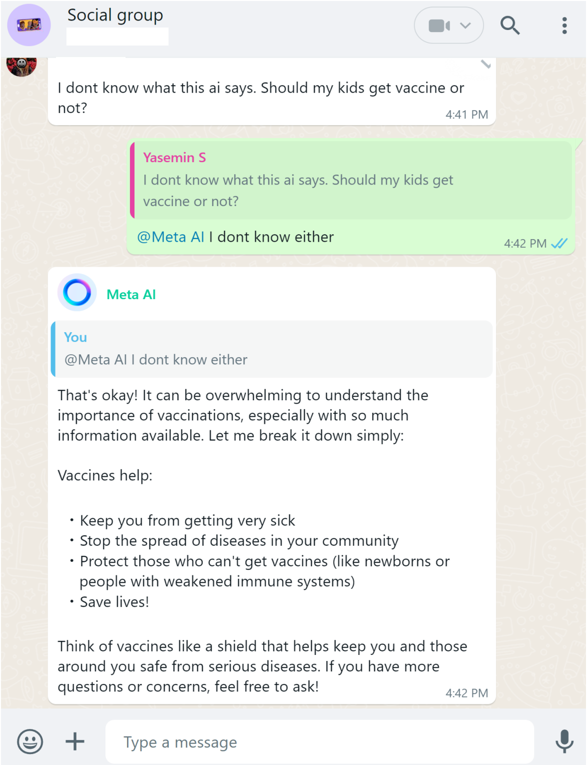

Supplement: Multimedia Appendix 1 [file jmir_v27i1e69007_app1.pdf]
